# Atopy patch tests may identify patients at risk for systemic contact dermatitis

**DOI:** 10.1002/iid3.280

**Published:** 2019-12-11

**Authors:** Suwimon Pootongkam, Sonia A. Havele, Hanna Orillaza, Eli Silver, Douglas Y. Rowland, Susan T. Nedorost

**Affiliations:** ^1^ Department of Dermatology University Hospitals Cleveland Medical Center, Case Western Reserve University Cleveland Ohio; ^2^ Case Western Reserve University School of Medicine Cleveland Ohio; ^3^ Division of Allergy and Immunology University Hospitals Cleveland Cleveland Ohio; ^4^ Department of Population and Quantitative Health Sciences (PQHS) Case Western Reserve University School of Medicine Cleveland Ohio; ^5^ Present address: Suwimon Pootongkam, Department of Dermatology Chulalongkorn University Bangkok Thailand; ^6^ Present address: Hanna Lucero, Section of Dermatology, Philippine General Hospital University of the Philippines Manila Philippines

**Keywords:** atopic dermatitis, atopy patch tests, elimination diets, protein contact dermatitis, systemic contact dermatitis

## Abstract

**Background:**

A subset of patients with positive patch tests demonstrates systemic contact dermatitis (SCD) upon ingestion or inhalation of the allergen. Concern has been raised about the use of patch tests for protein allergens (APTs) to detect SCD in atopic dermatitis (AD) patients.

**Methods:**

We present atopy patch test (APT) data for 97 people. We reviewed APTs and tests for antigen‐specific immunoglobulin E (IgE) to the same allergen in pediatric AD patients. We compared the frequency of APTs as a function of age in AD patients. To study the irritancy potential of APTs, we prospectively tested consenting non‐AD dermatitis patients undergoing evaluation for allergic contact dermatitis and healthy controls to an APT panel.

**Results:**

APT demonstrated fewer positive results than serum‐specific IgE or skin prick tests to the same allergen. Positive APT to food was more common in children under 3 years, whereas positive APT to aeroallergens were more common in teens and adults. Only positive APTs to dust mite were significantly more common positive in subjects without AD.

**Conclusion:**

Our aggregate findings suggest that most APTs, but not dust mite, behave like conventional patch tests to low‐potency allergens. They are more likely to be positive in patients with chronically inflamed skin and to identify allergens that cause SCD. The higher prevalence of APT positivity to foods in young children is consistent with food allergy as a trigger of AD (also known as SCD) being more common in children than adults. Positive APTs define patients who may have SCD; negative APTs may guide elimination diets.

## Graphical abstract

Atopy patch tests (APTs) indicate cutaneous sensitization to proteins, lower potency sensitizers more common in patients with impaired barrier. Positive APTs to food are more common in young children than adults. In some children with positive patch tests, dermatitis flares with ingestion of the allergen; this is known as systemic contact dermatitis. Children with dermatitis and without anaphylactic history may benefit from APTs to guide elimination diets.

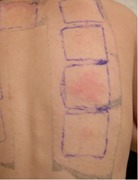

**Abbreviations**
ACD: allergic contact dermatitis
AD: atopic dermatitis
APT: atopy patch test
sIgE: serum‐specific IgE
SPT: skin prick tests

## 1 INTRODUCTION

Conventional patch tests identify contact allergens that are associated with systemic contact dermatitis (SCD) in a subset of patients with allergic contact dermatitis (ACD). SCD is defined as reactivation of dermatitis by ingestion or inhalation of the allergen that caused cutaneous sensitization.^1^ Likewise, atopy patch tests (APTs) detect delayed‐type hypersensitivity to proteins in contact with the skin in patients with atopic dermatitis (AD), and a subset of AD patients experience protein‐reactive SCD (pSCD), or flares of dermatitis upon ingestions or inhalation.^2^

In contrast, skin prick (SPT) and serum‐specific IgE (sIgE) tests detect immediate‐type hypersensitivity. These tests are useful to identify food allergy of the immediate type such as contact urticarial or anaphylaxis. These tests are used in the United States to evaluate patients with AD as well, despite the lack of mechanistic indication.

Recent literature suggests that cutaneous sensitization in patients with AD leads to SCD upon ingestion.^3^ An example of this sequence of immunological events is as follows: (a) infants with AD develop perioral irritant dermatitis from barrier dysfunction and drooling. (b) Initial exposure to a food protein on this inflamed skin causes sensitization. (c) This leads to a persistent Th2 response, as well as a less persistent Th1 response, compared with healthy controls.^4^

Conventional patch tests to compositae resin can identify patients who may develop dermatitis after the ingestion of related foods.^5^ Likewise, balsam of Peru patch testing has been used to diagnose food triggers of dermatitis, including tomato.^6^ Positive APT to cow milk in infants is associated with delayed‐type reactions to oral challenge.^7^ A previous study of patients aged 14 or older with a history of AD showed that longer the delay between ingestion of allergenic foods and elicitation of symptoms, the more severe the AD.^8^

In a systematic review of studies using APT vs double‐blind placebo‐controlled or open‐food challenges, APT was more useful in patients with AD and gastrointestinal symptoms than in those suspected of any type of food allergy as a whole. The pooled sensitivity in AD was 53.60% (95% CI: 51.10%‐56.00%) and the specificity was 88.60% (95% CI: 87.10%‐90.00%).^9^

We hypothesized that because cutaneous sensitization is less durable in atopic patients, APT would be less commonly positive than tests for immediate‐type hypersensitivity. We retrospectively reviewed pediatric patients we had tested with APT who also had results for SPT or sIgE available to the same antigen.

We hypothesized that APT results would correlate demographically with triggers associated with AD at various patient ages. We studied this retrospectively in all adults and children we had tested for clinical indication to food and pollen antigens.

Concern has been raised about irritancy and lack of standardization.^10^ APT to dust mite has been shown to be commonly positive in non‐AD patients.^11^ We, therefore, also prospectively tested consenting patients with nonatopic active dermatitis undergoing testing for ACD, as well as healthy controls with no inflammatory skin disease, to assess the number of positive APTs in non‐AD patients.

## 2 METHODS

In total, we tested 97 subjects with APT. We studied APTs in four cohorts. IRB approvals were obtained for this study from our hospital: #02‐12‐30 for chart review of test results in patients with AD in cohorts 1 and 2, and #04‐13‐11 for patients with non‐AD and healthy controls who provided written consent for prospective testing with a panel of APTs that were not chosen or indicated based on clinical history.

Cohorts 1 and 2 had AD defined as childhood onset flexural dermatitis, and the patch tests placed were driven by clinical history raising suspicion for the allergen.

### 2.1 Cohort 1

Thirty‐six pediatric patients with AD and documented APTs and SPT or sIgE to the same allergens based on clinical history were studied by a retrospective chart review (age range: 6 months to 17.6 years [mean ± SD = 7.3 ± 4.9]; 18 males and 18 females).

### 2.2 Cohort 2

Twenty‐six pediatric patients without documented SPT or sIgE and young adult AD patients, who had APT for clinical indication, were studied by retrospective chart review (age range: 6 months to 27 years [mean ± SD = 8.7 ± 6.2]; 12 males and 14 females).

### 2.3 Cohort 3

Twenty‐four patients with suspected contact dermatitis but without AD history, with (group 3a) or without (group 3b) rhinitis or asthma presenting for conventional patch testing for evaluation of ACD, tested after informed consent to a panel of APTs without a high degree of clinical suspicion (age range: 13.7 to 78.3 years [mean ± SD = 49.6 ± 17.5]; 11 males and 13 females).

### 2.4 Cohort 4

Eleven healthy adult control patients without any history of dermatitis or respiratory atopy were tested to a panel of APTs after providing informed consent.

SPTs were read 15 minutes following allergen exposure, and results were assessed by a board‐certified allergist. A wheal greater than or equal to 3 mm without reaction of negative control indicated a positive result. Positive and negative control tests were performed with histamine dihydrochloride 10 mg/mL and 0.9% NaCl solution, respectively. Grass mix and tree mix was used for SPT and compared with ryegrass/bluegrass and birch for APT, respectively. The IgE was detected using the ImmunoCAP system. sIgE detected above 0.35 kU/L was considered positive.

Cow's milk, egg white, and soy APTs were prepared by mixing 1 part commercial powder with 10 parts water to achieve approximately 6000 protein nitrogen units.^12^ Oat, wheat, corn, ryegrass/bluegrass, birch, dust mite, and ragweed were commercially obtained (Greer, NC) Dust mite from Chemotechnique (Malmo, Sweden) was used, and dandelion was used instead of Compositae mix Chemotechnique in cohorts 3 and 4.

Antigens in 12 mm Finn chambers (SmartPractice, AZ) were placed on uninvolved skin for 48 hours with readings at 48 to 72 hours after application.^5^ Reactions greater than or equal to 1+ were considered positive. We defined a 1+ reaction as erythema and four or more papules. Rim reactions were considered irritant, and reactions with three or fewer papules were considered doubtful and not counted as positive tests. This is a more quantitative modification of the European Task Force on Atopic Dermatitis 2003 consensus on grading APTs where a 2+ reaction was defined as a “few papules.”^12^

APT to foods was performed only if a patient had previously consumed that food without symptoms of immediate‐type allergy.

McNemar test was calculated for concordance/correlation of APT and SPT for food and inhalant allergens of the same patients. Individual and aggregate groups of food and aeroallergen APTs were compared by analyzing fractions of patients with positive APT to each antigen using Fisher's exact test.

## 3 RESULTS

**Figure 1:**
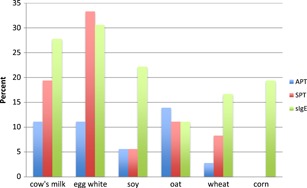
Percentage of positive APT, SPT, and sIgE to food allergens (cohort 1). There was significant discordance between APT and SPT to egg white (7/17; *P* < .05). There was also significant discordance between APT and sIgE to cow's milk (7/14; *P* < .05), egg white (6/14; *P* < .05), and wheat (5/11; *P* < .05). SPT and sIgE were not significantly discordant for any food allergen. Corn APT was not performed in any cohort 1 patients due to lack of clinical suspicion for allergy. APT, atopy patch test; sIgE, serum‐specific IgE; SPT, skin prick tests.

Results showed that APTs were less commonly positive than SPT or sIgE in children with AD (Figure 1). There was significant discordance between APT and SPT to the allergens as noted in the legend.

**Figure 2:**
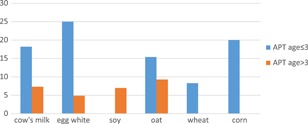
Percentage of positive APT to food allergens in age less than or equal to 3 and age greater than 3 (cohorts 1 and 2; *P* = .08). APT, atopy patch test.

**Figure 3:**
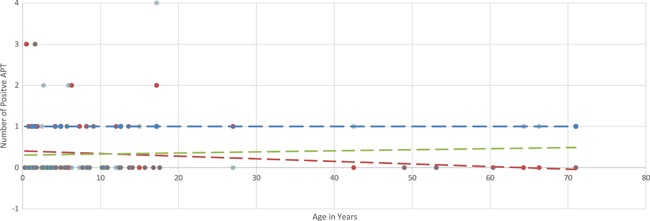
Atopic dermatitis patients showing number of positive atopy patch tests (APTs) by age. Trend lines superimposed on raw data: red line, food APTs (cow's milk, oat, soy, egg, wheat); green line, aeroallergen APTs (birch, ragweed, grass, and compositae resin); blue line, dust mite. AD, atopic dermatitis; APT, atopy patch test.

**Figure 4:**
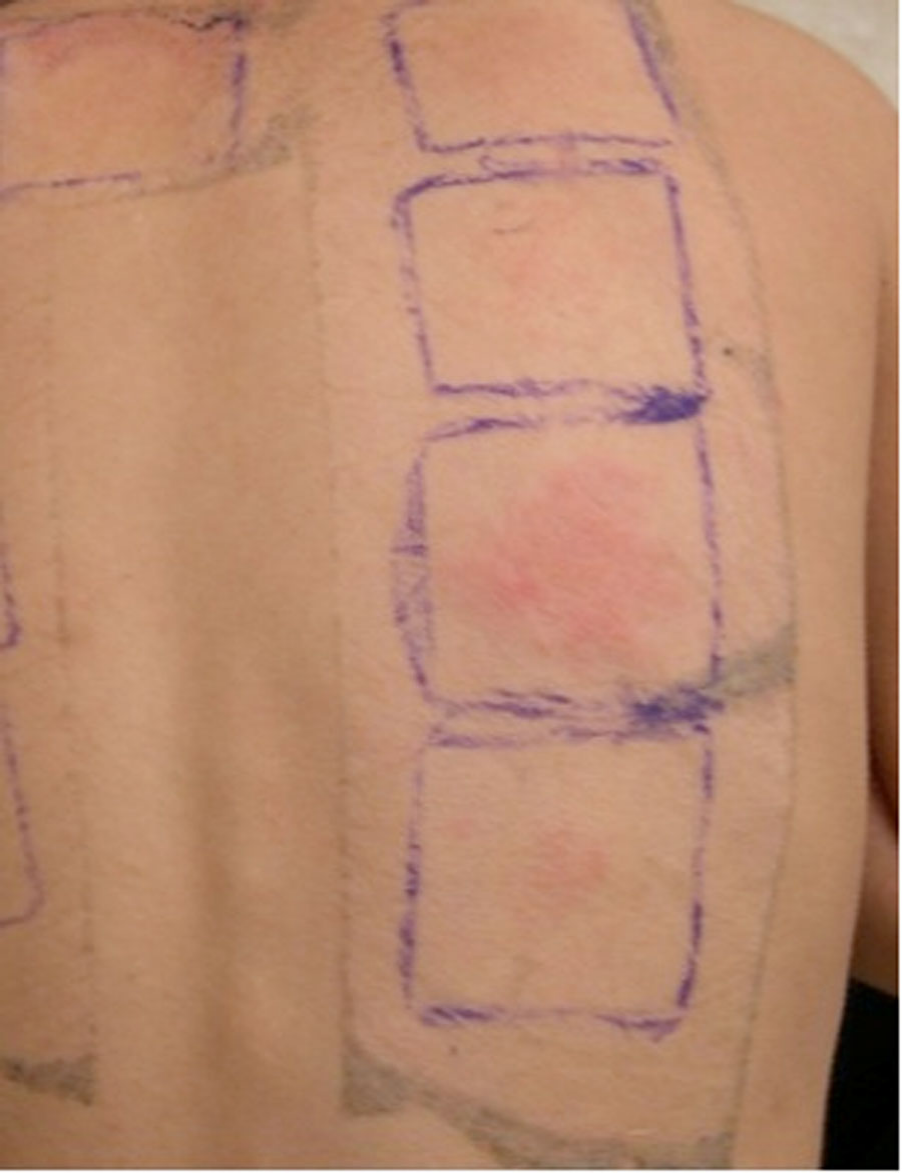
Positive atopy patch test (APT) (middle chamber of three full chambers in column shown) to birch in a child with springtime flares. The dust mite APT at the bottom of the column of tests was considered weakly positive

Positive tests to foods occurred more frequently in children 3 years or younger (*P* = .08) (Figure 2) whereas in older children and adults, APTs to aeroallergens were more common than food allergens (Figures 3 and 4).

**Figure 5:**
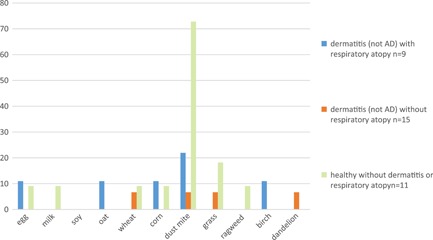
APT reactions in healthy controls defined as no history of dermatitis or respiratory atopy and dermatitis patients without a history of childhood onset flexural dermatitis (non‐AD) with or without respiratory atopy. AD, atopic dermatitis; APT, atopy patch test.

Of the 11 healthy controls, 8 had positive APTs to dust mite, 2 to bluegrass, and 1 each to cow's milk, corn, egg, wheat, and ragweed, with a single subject accounting for the last four reactions (Figure 5). Dust mite APT was more likely to be positive in dermatitis patients without atopy and in healthy controls (groups 3b and 4) than in patients with atopy (groups 1, 2, and 3a) *P* = .013.

## 4 DISCUSSION

Our study is one of the largest of APTs and has the strengths of a single center, with both pediatric and adult AD cohorts and comparator cohorts of non‐AD dermatitis patients and healthy controls. The aggregate findings suggest that APTs behave like conventional patch tests to low‐potency allergens such as propylene glycol, tocopherol, and parabens. They are more commonly positive in patients with chronically inflamed skin such as AD patients or hand dermatitis patients who do wet work^13^ and are often associated with SCD.^14^

We know that positive conventional patch tests are not specific for SCD; SCD occurs in only a subset of patients, often those with a history of AD. Likewise, positive APTs indicate that the subject has been sensitized to the allergen, but only a subset of these subjects will demonstrate SCD. The presence of CLA + T cells may be required to elicit SCD in addition to the T memory cells that are required to elicit ACD in the context of the patch test.^15^ Negative APTs have potential value in reducing the number of possible culprit food proteins and may prevent unnecessary dietary restriction.

Except for dust mite, APTs do not appear to be irritant or commonly positive in healthy controls. Potent sensitizers in the healthy population tend to have strong irritant properties, and some of the dust mite reactions may represent true allergy to this potent allergen. AD patients are less likely to react to potent allergens (eg, poison ivy). In contrast, AD patients are more likely to react to weakly potent allergens,^13^ including those known to cause SCD.^14^

Aeroallergens are very difficult to avoid and therefore APTs to aeroallergens are of less utility than those to foods. However, previous research in adults has demonstrated the benefit of avoiding foods that cross‐react with aeroallergens.^16^

In a recent study, pruritic dogs suspected of food‐triggered dermatitis underwent a washout interval consuming a diet confined to new foods and were then rechallenged with the individual foods previously suspected of causing symptoms. Sensitivity and specificity of APTs to the suspected foods were 96.7% and 89.0% compared with 6.7% and 91.4% for food‐specific IgE. The negative predictive value was 99.3 and the positive predictive value was 63.0%.^17^ The authors concluded that the patch tests were most useful to identify foods that did not need to be included in elimination trial diets.

We frame APTs in exactly the same way that we interpret results of conventional patch tests. Not every positive test indicates a relevant current causative factor for dermatitis, and only a subset of patients with a positive patch test will have SCD. SCD involves a Th2 response,^18^ so it is likely that patch tests in AD patients (ie, APTs) may be even more likely to identify triggers of SCD.

There are limitations to our study. We did not perform oral challenge because washout with only new foods, followed by rechallenge with foods yielding a positive APT and observation over a period of days as performed in dogs, is very difficult in humans. Therefore, we do not have data on the incidence of SCD in our cohort. We have observed improvement in dermatitis with food avoidance guided by APT and confirmed by rechallenge in some patients. However, clinical improvement is confounded in clinical practice by additional therapeutic measures such as bleach baths to treat for sensitization to commensal organisms and medications such as corticosteroids and calcineurin inhibitors.

Cautions include the unknown optimal interval between APT studies for determining continued positivity. There is also concern that food avoidance might lead to a more severe immediate‐type hypersensitivity reaction upon rechallenge.

The group most likely to benefit from APTs is children with refractory dermatitis and without a history of anaphylactic symptoms, regardless of sIgE results. Patch tests detect cutaneous sensitization, and a subgroup of patients with positive patch tests will have SCD. Negative patch tests to foods may narrow elimination diets.

## CONFLICT OF INTERESTS

The authors declare that there are no conflict of interests.

## DATA AVAILABILITY STATEMENT

The data that support the findings of this study are available from the corresponding author upon reasonable request.

## ETHICS STATEMENT

Institutional Review Board at our hospital provided ethical approval for this human subjects research in compliance with the US Federal Policy for the Protection of Human Subjects.

## References

[1] Rietschel RL, Fowler JF Jr, eds. Systemic contact dermatitis Fisher's Contact Dermatitis. 6th ed. Hamilton, ON: BC Decker Inc; 2008.

[2] Mansouri M , Rafiee E , Darougar S , Mesdaghi M , Chavoshzadeh Z . Is the atopy patch test reliable in the evaluation of food allergy‐related atopic dermatitis? Int Arch Allergy Immunol. 2018;175(1‐2):85‐90. 29332097 10.1159/000485126

[3] Matsumoto K , Mori R , Miyazaki C , Ohya Y , Saito H . Are both early egg introduction and eczema treatment necessary for primary prevention of egg allergy? J Allergy Clin Immunol. 2018;141(6):1997‐2001. 29522845 10.1016/j.jaci.2018.02.033

[4] Newell L , Polak ME , Perera J , et al. Sensitization via healthy skin programs Th2 responses in individuals with atopic dermatitis. J Invest Dermatol. 2013;133(10):2372‐2380. 23528819 10.1038/jid.2013.148

[5] Paulsen E . Systemic allergic dermatitis caused by sesquiterpene lactones. Contact Dermatitis. 2017;76(1):1‐10. 27568784 10.1111/cod.12671

[6] Salam TN , Fowler JF Jr . Balsam‐related systemic contact dermatitis. J Am Acad Dermatol. 2001;45(3):377‐381. 11511833 10.1067/mjd.2001.114738

[7] Majamaa H , Moisio P , Holm K , Kautiainen H , Turjanmaa K . Cow's milk allergy: diagnostic accuracy of skin prick and patch tests and specific IgE. Allergy. 1999;54(4):346‐351. 10371093 10.1034/j.1398-9995.1999.00834.x

[8] Celakovska J , Bukac J , Ettler K , Ettlerova K , Krcmova I . Atopic dermatitis in adolescents and adults‐the evaluation of association with other allergic diseases and parameters. Food Agric Immunol. 2017;28:933‐948.

[9] Luo Y , Zhang GQ , Li ZY . The diagnostic value of APT for food allergy in children: a systematic review and meta‐analysis. Pediatr Allergy Immunol. 2019;30(4):451‐461. 30703250 10.1111/pai.13031

[10] Boyce JA , Assa'ad A , Burks AW , et al. Guidelines for the diagnosis and management of food allergy in the United States: summary of the NIAID‐Sponsored Expert Panel report. J Am Acad Dermatol. 2011;64(1):175‐192. 21167411 10.1016/j.jaad.2010.11.020

[11] Silverberg JI , Hanifin JM , Law S , White K , Storrs FJ . Lack of association between dust mite sensitivity and atopic dermatitis. Dermatitis. 2016;27(2):59‐67. 26983092 10.1097/DER.0000000000000165

[12] Lachapelle J‐M , Maibach HI . Patch Testing and Prick Testing. Berlin, Heidelberg: Springer‐Verlag; 2003.

[13] Kohli N , Nedorost S . Inflamed skin predisposes to sensitization to less potent allergens. J Am Acad Dermatol. 2016;75(2):312‐317. 27287247 10.1016/j.jaad.2016.03.010

[14] Scott JF , Conic RRZ , Kim I , Rowland DY , Nedorost ST . Atopy and sensitization to allergens known to cause systemic contact dermatitis. Dermatitis. 2019;30(1):62‐66. 30640765 10.1097/DER.0000000000000436 PMC6334658

[15] Jensen CS , Lisby S , Larsen JK , Veien NK , Menné T . Characterization of lymphocyte subpopulations and cytokine profiles in peripheral blood of nickel‐sensitive individuals with systemic contact dermatitis after oral nickel exposure. Contact Dermatitis. 2004;50(1):31‐38. 15059101 10.1111/j.0105-1873.2004.00294.x

[16] Reekers R , Busche M , Wittmann M , Kapp A , Werfel T . Birch pollen‐related foods trigger atopic dermatitis in patients with specific cutaneous T‐cell responses to birch pollen antigens. J Allergy Clin Immunol. 1999;104(2 pt 1):466‐472. 10452773 10.1016/s0091-6749(99)70395-7

[17] Bethlehem S , Bexley J , Mueller RS . Patch testing and allergen‐specific serum IgE and IgG antibodies in the diagnosis of canine adverse food reactions. Vet Immunol Immunopathol. 2012;145(3‐4):582‐589. 22301200 10.1016/j.vetimm.2012.01.003

[18] Jacob SE , Sung CT , Machler BC . Dupilumab for systemic allergy syndrome with dermatitis. Dermatitis. 2019;30(2):164‐167. 30829809 10.1097/DER.0000000000000446

